# Integrated mRNA and miRNA Omics Analyses Reveal Transcriptional Regulation of the Tolerance Traits by *Aquatica leii* in Response to High Temperature

**DOI:** 10.3390/insects16030316

**Published:** 2025-03-18

**Authors:** Chao Liu, Jiapeng Li, Lihong Yan, Yuting Zhu, Zikun Li, Chengquan Cao, Yiping Wang

**Affiliations:** 1 College of Forestry and Biotechnology, Zhejiang Agricultural and Forestry University, Lin’an 311300, China; wa88711007@163.com (C.L.); yanlihong2023@163.com (L.Y.); z1960442236@163.com (Y.Z.); fclzk123@163.com (Z.L.); 2 School of Management, Chengdu University of Information Technology, Chengdu 610225, China; 3 College of Life Sciences, Leshan Normal University, Leshan 614004, China; chqcao1314@163.com

**Keywords:** aquatic firefly, transcriptome, miRNA, heat stress, high temperature, global warming

## Abstract

Within the context of global warming, understanding the molecular mechanisms behind physiological plasticity and local adaptation is essential for insect populations. This study performed an integrated miRNA and mRNA analysis on *Aquatica leii* larvae exposed to temperatures of 20 °C, 24 °C, 28 °C, and 32 °C. Under varying thermal conditions, 1983 genes exhibited differential expression (i.e., DEGs). These genes showed significant enrichment in Kyoto Encyclopedia of Genes and Genomes (KEGG) pathways related to carbohydrate metabolism, glycan biosynthesis and metabolism. Notably, we detected that the “neuroactive ligand–receptor interaction” signaling pathway, which is involved in environmental information processing, was significantly upregulated in the 28 °C and 32 °C treatment groups. This indicates that starting at 28 °C, *A. leii* needs to maintain normal cellular physiological functions by regulating ligand–receptor binding and signal transduction. Furthermore, 220 differentially expressed miRNAs (DEMs) were detected under the different temperature treatment conditions. An interaction network was constructed between key DEMs and DEGs, revealing 12 significant DEM-DEG regulatory pairs in *A. leii* under different temperature treatments. We found three miRNA-mRNA candidate modules that could be involved in *A. leii*’s response to high temperature, including ggo-miR-1260b and ptr-miR-1260b/RN001_010114, CM069438.1_43851/RN001_014852, and CM069438.1_43851/RN001_014877. Our data provide deeper insights into the molecular responses of *A. leii* to the high temperature at the miRNA and mRNA levels.

## Simple Summary

As worldwide warming intensifies, understanding how insect populations adapt to rising temperatures has been an important scientific question. The survival and reproduction of insects depend on their physiological adaptability to environmental changes. We studied *Aquatica leii* larvae to explore their molecular responses to temperature changes. We simulated different temperature conditions (20 °C, 24 °C, 28 °C, and 32 °C) to examine changes in their mRNA and microRNAs expression. Our research has found that under high-temperature conditions of 28 °C and 32 °C, there are significant changes in gene expression in *A. leii*, involving key physiological processes such as carbohydrate metabolism and glycan biosynthesis. In addition, our findings indicate that the “neuroactive ligand–receptor interaction” pathway is significantly activated at high temperatures, implying that *A. leii* may maintain normal cellular functions by regulating ligand–receptor binding. We also identified 220 differentially expressed microRNAs and constructed regulatory networks between these miRNAs and genes, revealing potential molecular regulatory mechanisms in insects under high-temperature conditions. Our study provides a new perspective on how insects survive in high-temperature environments, which is of great significance for protecting insect biodiversity and predicting their adaptability to climate change.

## 1. Introduction

A hot topic of conservation biology is the examination of the ways in which species adapt beyond their current physiological limitations in the context of global climate change [1,2]. Physiological change enhances the ability of organisms to cope with environmental change, serves as a basis for evolutionary adaptation and population persistence, and is necessary for evolutionary rescue during environmental change [1,3,4]. Whether long-term or short-term adaptation, a comprehensive understanding of the underlying molecular mechanisms involved in physiological plasticity or local adaptation is critical to accurately predict species’ responses to climate change [1,5].

Climate change has caused global warming and increased the frequency of extreme weather events. Temperature, a key abiotic factor affecting animal physiology, is recognized as a fundamental manifestation of global warming [6]. Insects, as the most diverse group of ectothermic animals, are more sensitive to global warming due to their dependence on external heat sources for body temperature regulation [7,8]. This is especially the case with regard to some aquatic insects, which are dependent on water temperature for body temperature regulation and therefore have to adapt to fluctuations in this parameter through biochemical, physiological, and morphological flexibility, as well as microevolutionary changes [3,9,10].

Insect species are extremely threatened by global warming; numerous studies indicate that high-temperature stress can cause heat injury in insects, leading to alterations in their morphology, cellular structure, biochemistry, molecular biology, physiology, and even impacting their endosymbionts [6,11,12,13]. As a result of the long process of natural evolution, insects have evolved several adaptation strategies to cope with high-temperature, including behavioral characteristics, phenotypic plasticity, genetic adaptation, physiological and biochemical levels, as well as in thermoregulation [3,6,13,14]. For example, studies have demonstrated that a 1 °C increase in temperature can lead to a reduction in body size of 1–3% in beetles [15]. The proteomic data from *Drosophila melanogaster* indicates an increase in the expression of proteasome proteins during periods of high-temperature stress, which accompanied by a decrease in the activity of certain basal metabolic functions [3]. Additionally, the heat shock protein (*HSP*)-mediated stress response represents a crucial strategy for the management of high-temperature in insects [16,17]. *HSP* are expressed in perineurium, glial cells, and nerve membranes, initiating a stress response to protecting the nervous system from high temperatures when insects are exposed to elevated temperatures [18]. In natural habitats, aquatic insects are exposed to more complex high-temperature environments than terrestrial insects, (e.g., hypoxia and salinity stress) [19]. Previous studies documented the direct impact of high water temperature on physiology of aquatic insects [19]. Prolonged warm water exposure can lead to a decrease in the body size of aquatic insects [20]. It was also shown that short-term exposure to high temperatures could reduce the longevity of *Heterelmis comalensis*, an aquatic insect with vestigial wings and limited dispersal ability [21]. Furthermore, it was shown that in some stonefly species, gene expression is under the influence of changes in water temperature, in which respiration-related and metabolic-related genes may serve important roles in high-temperature adaptation and could be utilized in biomonitoring studies [1]. Although these findings are encouraging, and insect thermal biology is well-researched, aquatic insects continue to receive little attention. For fireflies, much attention has focused on their luminescence, while there is a significant lack of data regarding the response of aquatic fireflies to temperature change.

Fireflies (Lampyridae), known as luminous beetles (Coleoptera), are widely distributed in temperate, subtropical, and tropical zones [22,23]. The distinctive bioluminescent courtship displays of fireflies have attracted tourists, nature lovers, and various researchers, making them among the most charismatic beetles [24,25]. Currently, over 2200 firefly species have been described worldwide, exhibiting a wide range of life history traits and extensive ecological diversity [26,27,28]. Interestingly, species in this family occupy a wide range of niches, including those in terrestrial, aquatic, and semi-aquatic habitats [25,27]. At present, most studies on fireflies have mainly focused on their taxonomy, bioluminescence, courtship behavior and biology [24,29,30]. However, global warming is affecting the growth, development, and distribution of fireflies in different ways. Therefore, achieving a better understanding of the mechanisms involved in the high-temperatures response of fireflies is timely and essential.

*Aquatica leii* is a species of aquatic fireflies unique to China, which was originally discovered and described by Fu and Ballantyne in 2006, with its taxonomic status revised in 2010 [31,32]. Its populations are currently known as one of the largest breeding populations of aquatic fireflies in southern China [31,32]. To date, numerous scientists have explored the metabolic and molecular adaptations of *A. leii* to freshwater environments, as well as the effects of benzo(a)pyrene stress on miRNAomic and mRNA transcriptional profiles in *A. leii* [33,34,35,36]. However, no studies have been reported on the molecular mechanism response of this species to high temperatures. Therefore, our study employed an integrated transcriptomic and miRNAomic approach to analyze mRNA and miRNA expression profiles in sixth instar larvae subjected to different temperature treatments. By conducting a comprehensive miRNA-mRNA integrated analysis, we aimed to identify key regulatory networks and molecular pathways involved in the thermal stress response. In summary, our findings could help us elucidate the mechanisms of high-temperature stress in *A. leii* and provide new possibilities for biomonitoring using key genes. Our research aims to combine theory and practice, providing a scientific basis for artificial breeding, particularly in terms of temperature control for *A. leii*.

## 2. Materials and Methods

### 2.1. Insect Materials and Rearing Conditions

Sixth instar larvae of *A. leii* (1.0 ± 0.2 cm) were sourced from the Culture Centre of Fireflies in Ganzhou, Jiangxi Province, China, and maintained at the College of Forest and Biotechnology, Zhejiang Agricultural and Forestry University, Zhejiang, China A total of 500 sixth instar larvae were randomly selected and acclimated by being transferred to tanks sized 50 × 40 × 15 cm, with 4 L of well-dechlorinated tap water (with a temperature of 22 ± 1 °C, pH 6.5–7.5, and dissolved oxygen 7.0 ± 0.3 mg/L). This preconditioning phase lasted 7 days prior to formal experimentation. During the acclimation and experimental periods, *A. leii* larvae were fed Chinese Mystery Snail (*Cipangopaludina chinensis*) once every two days, with each feeding providing approximately 25 g of snail meat for every 100 larvae, see also [27,31].

### 2.2. High-Temperature Treatment and Sample Collection

The environmental temperature treatment simulated the natural daily temperature changes in a day–night mode, with the initial temperature set at 20 °C based on local summer temperature observations and previous research (Table S1) [37,38]. The high-temperature treatments were set at 24 °C, 28 °C, and 32 °C based on the summer temperatures in Hangzhou, Zhejiang Province, China, and future global warming scenarios [39,40]. For elevated temperature treatments, the water temperature increase begun at 9:00 a.m. using heating and was stopped at 4:00 p.m., as previously described [38]. In addition, the duration of the high-temperature treatments was set at 7 days, which is close to the current average duration of recorded lake heatwaves of 7.7 ± 0.4 days [41]. However, sustained high temperatures inevitably bring about anoxic stress that may affect the experiment, and in order to minimize experimental errors we added an oxygen pump to the water. A total of 30 individual *A. leii* larvae samples were separately collected from different high-temperature treatments and a control group. Each treatment group included three biological replicates, with 10 larvae pooled per repetition to decrease inter-individual variation [33,34]. The experiments were conducted in triplicates for each treatment. The samples were promptly frozen in liquid nitrogen and stored at −80 °C for later RNA extraction.

### 2.3. RNA Isolation

Total RNA was isolated with the TRIzol™ Plus RNA Purification Kit (Invitrogen, Carlsbad, CA, USA) according to the manufacturer’s guidelines. The RNA integrity and DNA contamination were confirmed in 1.5% agarose gel electrophoresis, as described previously [34,42]. RNA purity and concentration were measured using a NanoPhotometer spectrophotometer (Implen GmbH, München, Germany) and a Qubit2.0 Fluorometer (Thermo Fisher, Waltham, MA, USA), while RNA integrity was assessed with an Agilent 2100 Bioanalyzer (Agilent Technologies, Santa Clara, CA, USA).

### 2.4. mRNA Sequencing and Genomes Align

Following total RNA extraction, *A. leii* sequencing libraries were prepared using standard Illumina protocols [36,43]. The library was sequenced using the Illumina Novaseq 6000 platform to generate 150 bp paired-end reads. Raw sequencing reads were quality controlled using FastQC (v.0.11.9) and processed with Fastp (v.0.19.5) to remove adaptors and low-quality sequences, resulting in clean reads [44]. The reference genome index of *A. leii* (NCBI accession number: GCA_035610365.1) [24] was constructed using HISAT2 (v.2.1.0) [45]. Paired–end clean reads were aligned to this genome in the orientation mode, and the mapped reads were assembled with StringTie [46]. mRNA sequencing data were deposited in the NCBI SRA (https://www.ncbi.nlm.nih.gov/sra/ (accessed on 20 December 2024) (PRJNA1200824).

### 2.5. Screening of Differentially Expressed Genes (DEGs) and KEGG Enrichment Analysis

The gene expression levels for the differential analysis were calculated using the fragments per kilobase of transcript per million mapped fragments (FPKM) method with RSEM software (v.1.3.3) [47]. Significant differentially expressed genes (DEGs) were identified using the DESeq2 R package [48], with criteria of |log_2_FoldChange| > 1.0 and a Benjamini–Hochberg adjusted *p*-value (Padj) ≤ 0.05, as previously described in [33,36]. DEGs were visualized through heatmaps generated by the R heatmap package. The Mufzz method was employed to analyze the expression patterns of DEGs.

Gene Ontology (GO) enrichment and Kyoto Encyclopedia of Genes and Genomes (KEGG) pathway enrichment analysis of DEGs between different pairwise comparisons were performed respectively using Cluster Profiler [49]. The results of the enrichment analyses are visualized using the ggplot2 package in R language and SRplot online platform (https://www.bioinformatics.com.cn/srplot accessed on 1 December 2024) [50].

### 2.6. Small RNA Sequencing and Genomes Align

After the total RNA was extracted, library preparations for the sRNA sequencing of *A. leii* were constructed using and Illumina VAHTS Small RNA Library Prep Kit (Vazyme, Nanjing, China) according to the standard steps provided by Illumina Company [51,52]. The constructed libraries were sequenced using Illumina Novaseq 6000 platform (Illumina, San Diego, CA, USA). In order to obtain high-quality clean reads for downstream analysis, Fastx_toolkit was used to filter low-quality reads, cut adapters and quality control raw reads. Then, clean reads were aligned with the Rfam database and NCBI GenBank database to classify and annotate the small RNAs, and to remove rRNA, scRNA, sonRNA, snRNA, and tRNA. The small RNAs were mapped on to the reference genome of *A. leii* (NCBI accession number: GCA_035610365.1) [24] with Bowtie2 (v.2.5.4) [53]. The miRNA sequencing data were deposited in NCBI SRA (https://www.ncbi.nlm.nih.gov/sra/ accessed on 20 December 2024) (PRJNA1200912).

### 2.7. Screening of Differentially Expressed miRNAs (DEMs)

To identify miRNAs in *A. leii*, the effective reads mapped to reference genomes were compared against precursor and mature miRNA sequences in miRBase v22 using miRDeep2 with default parameters [54,55], and the normalized expression value of the miRNAs were calculated according to the transcripts per million (TPM). The standards |log2FoldChange | > 1.0 and Padj ≤ 0.05 were used to determine significant differentially expressed miRNAs (DEMs), as previously described [34,56]. Mufzz (V.2.66.0) was used to analyze the expression patterns of the DEMs by the C-means method.

### 2.8. Prediction and Enrichment Analysis of DEMs Targets

Target genes of the DEMs were identified utilizing dedicated miRNA target gene prediction databases, such as miRanda [57]. To further understand the functional category of these target genes, we conducted an analysis of the GO terms and KEGG pathway using Cluster Profiler [49]. The statistical significance of the GO and KEGG enrichment analyses was evaluated using Fisher’s exact test, which was performed using the fisher.test() function in the R package, with a *p*-value of 0.05 as the threshold for significance.

### 2.9. Quantitative PCR (qPCR) Analysis

A total of 2 μg RNAs was extracted and reverse transcribed to cDNA using the PrimeScriptTM RT Reagent Kit (TaKaRa, Dalian, China) in accordance with the manufacturer’s instructions. qPCR was conducted using TB Green^®^ Premix Ex TaqTM II (TaKaRa, Dalian, China) in CFX96 Touch Deep Well Real-Time PCR Detection System (Bio-Rad, Hercules, CA, USA), as described previously [43]. For each candidate gene, *A. leii* ubiquitin-40S ribosomal protein S27Ae was used as an internal standard [42], and the relative expression levels in mRNA abundance were calculated using the 2^−ΔΔCT^ method [58]. The results represent the average of three biological replicates, each with two technical replicates each. The statistical significance of differences was calculated using Student’s *t*-test in Microsoft Excel, and a *p* value < 0.05 was considered statistically significant. The qPCR primers are detailed in Table S2.

## 3. Results

### 3.1. Overview of A. leii mRNA Sequencing Data

**Figure 1:**
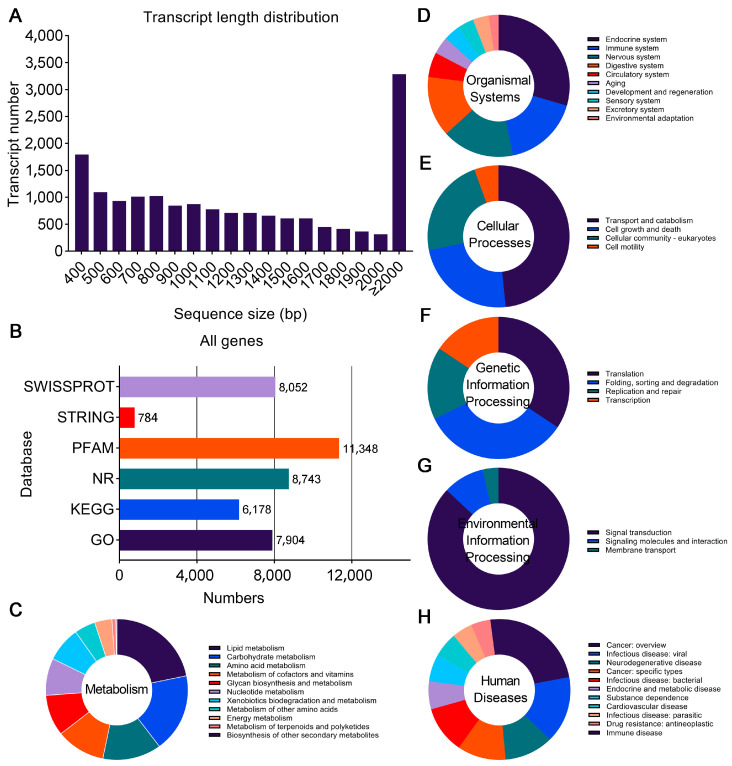
RNA-seq of *A. leii* following different temperature treatments: (**A**) basic parameters of the length distribution of the assembled transcripts of *A. leii*; (**B**) number of genes annotated via different databases, including Protein Family (Pfam), NCBI non-redundant protein (NR), Swiss-Prot, Gene Ontology (GO), Kyoto Encyclopedia of Genes and Genomes (KEGG), and Search Tool for Recurring Instances of Neighbouring Genes (STRING); (**C**–**H**) classification of annotated KEGG terms. A total of 7178 genes were annotated into 6 main KEGG terms.

To identify the expression profiles of *A. leii* responsive genes following different temperature treatments, we performed a transcriptome analysis using sixth instar larvae of *A. leii* exposed to different temperatures (20 °C, 24 °C, 28 °C, and 32 °C). A total of 70.80 Gb raw data were obtained from the 12 *A. leii* samples, with an average of 5.90 Gb of raw data per sample. After quality control, a total of 68.24 Gb of clean data were obtained, which produced approximately 5.69 Gb of clean data for each sample. The quality scores for the Q20 and Q30 levels of the clean data were more than 98.62% and 96.59%, respectively, the average GC content of the transcriptome was 39.20% (Table S3), indicating that the sequencing quality in this study was good. As shown in Figure 1A, the length of the assembled transcript was primarily greater than 2000 bp (3288 genes) in length with median and average lengths of 1086 bp and 1479 bp, respectively. Genes were annotated using multiple protein databases, in which the most significant number of *A. leii* genes were annotated in the Protein Family (Pfam) database (11,348 genes), NCBI non-redundant protein (NR) database (8743 genes), and Search Tool for Recurring Instances of Neighbouring Genes (STRING) database (8052 genes) (Figure 1B).

To understand the functional distribution of these genes, we performed functional classification based on the annotated GO terms and KEGG pathway. A total of 7904 (47.98% of all genes) genes were effectively assigned to at least one GO term (Figure 1B). In terms of biological process, the three most abundant terms were single-organism process (5714), cellular process (5256), and metabolic process (4293) (Table S4). For molecular function, proteins were mostly assigned to the binding (4994), catalytic activity (3817), and transporter activity (620) categories (Table S4). Within the cellular component category, the majority of the GO terms were predominantly assigned to cell (6643), cell part (6632), and organelle (5281) (Table S4). Furthermore, a total of 6178 genes (37.50% of all genes) were mapped onto KEGG pathways (Figure 1B), which included those related to metabolism, organismal systems, cellular processes, genetic information processing, and environmental information processing (Figure 1C,D). Taken together, these results suggest that different temperature treatments can lead to extensive gene rearrangements in *A. leii*.

### 3.2. Analysis of DEGs of A. leii Under Different Temperature Treatments

**Figure 2:**
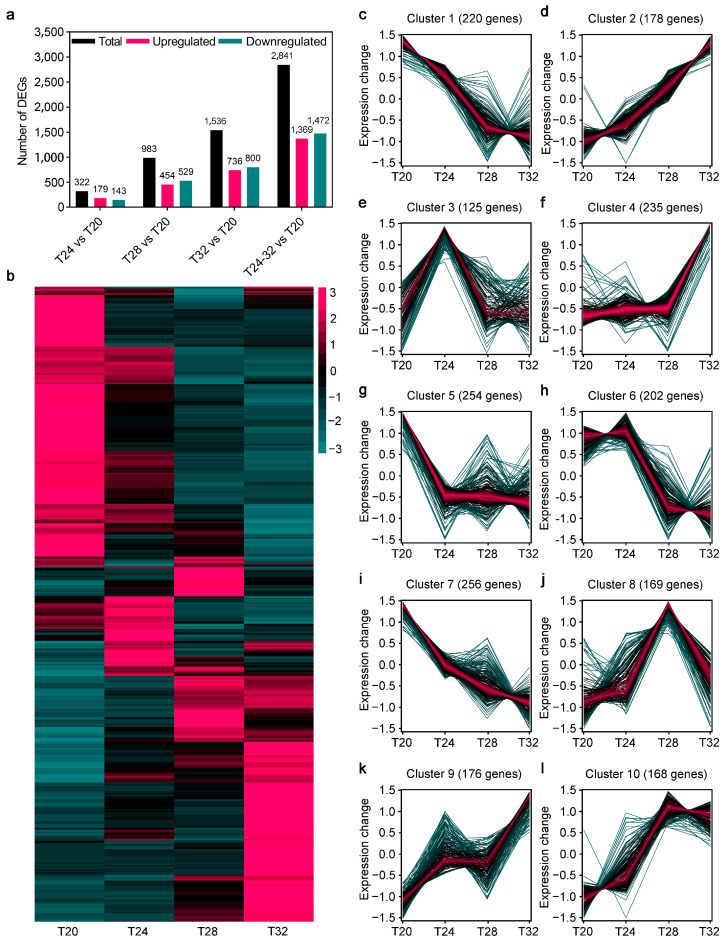
Analysis of DEGs under various temperature treatments in *A. leii*: (**a**) numbers of the total, upregulated, and downregulated DEGs in *A. leii* under various temperature (20 °C, 24 °C, 28 °C, and 32 °C) treatments; (**b**) expression profile heatmap of the DEGs identified in A. leii following temperature treatments at 20 °C, 24 °C, 28 °C, and 32 °C, with the heatmap scale ranges representing normalized expression levels, from low to high, on a log2 scale from −3 to 3; (**c**–**l**) mufzz clustering of the dynamic expression of DEGs in *A. leii* under various temperature treatments.

To determine the molecular mechanisms of *A. leii* under different temperature treatments, we compared the gene expression levels of *A. leii* following temperature treatments at 20 °C, 24 °C, 28 °C, and 32 °C. As shown in Figure 2a, 322 DEGs in the T24 vs. T20 comparison, including 179 upregulated and 143 downregulated DEGs; 983 DEGs in the T28 vs. T20 comparison, including 454 upregulated and 539 downregulated; and 1536 DEGs in the T32 vs. T20 comparison, including 736 upregulated and 800 downregulated DEGs, were identified in different temperature treatment of *A. leii* (Figure 2a). Then, we analyzed the expression pattern of these different temperature-responsive DEGs using a heatmap (Figure 2b). DEGs were subsequently grouped into 10 clusters based on the expression patterns (Figure 2c–l). Specifically, the hierarchical clustering analysis demonstrated that the expression patterns of Cluster 1 (220 genes) and Cluster 7 (1791 genes) DEGs were quickly downregulated by the temperature treatments, and these decreased expression patterns extended along the same trajectory (Figure 2c,i). In contrast, the expression patterns of Cluster 2 (178 genes) DEGs were rapidly induced and increased with T20 (Figure 2d), continuing along the same trajectory as the temperature rose. Additionally, Clusters 3, 4, 5, 6, 8, 9, and 10 presented different expression patterns under the various temperature treatments (Figure 2e–h,j–l). Together, these gene expression profiles suggest that there might be a specific response pattern of *A. leii* to different temperature treatments.

In addition, at T24, the DEGs were significantly enriched (i.e., pathways occurring more frequently than expected by chance) for oxidoreductase activity (*p* = 7.40 × 10^−6^ ), salivary gland histolysis (*p* = 2.00 × 10^−5^), and genitalia morphogenesis (*p* = 2.72 × 10^−5^) GO terms (Table S5); at T28, the DEGs are significantly enriched in oxidoreductase activity (*p* = 0 × 10 ^0^), and iron ion binding (*p* = 0 × 10 ^0^) GO terms (Table S6); and at T32, the DEGs are significantly enriched in monooxygenase activity (*p* = 0 × 10 ^0^), and iron ion binding (*p* = 0 × 10 ^0^) GO terms (Table S7).

### 3.3. KEGG Enrichment Analysis of Temperature-Responsive DEGs of A. leii

To further explore the adaptive strategy of *A. leii* to temperature, we conducted a KEGG enrichment analysis on the T24-, T28-, and T32-responsive DEGs and calculated the *p*-values using Fisher’s exact test for each KEGG pathway. For the T24 group, genes belonging to the “Glycosaminoglycan degradation” pathway were significantly downregulated, while those in the “Lysosome” pathway were significantly upregulated (*p* < 0.05) (Figure S1). For the T28 group, genes belonging to the “Metabolic pathways” and “Glycerolipid metabolism” pathways were significantly upregulated (*p* < 0.05) (Figure S2). For the T32 group, the genes belonging to the “Metabolic pathways” and “Fructose and mannose metabolism” pathways were significantly downregulated (*p* < 0.05) (Figure S3). In these pathways, the metabolic pathways were most prominent.

**Figure 3:**
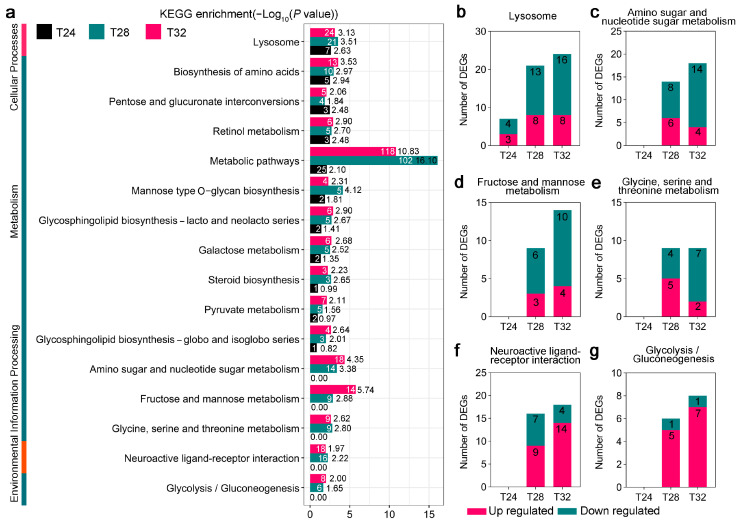
Effects of different temperature treatments on KEGG pathways of *A. leii*. (**a**) KEGG enrichment analysis of DEGs of *A. leii* from the T24, T28, and T32 group or T28 and T32 group. The abscissa presents the statistical significance (−Log10(*p* value)) of the KEGG terms under different temperature treatments in *A. leii*. Black, teal, and Pink represent T24, T28 and T32, respectively. Numbers in white represent the number of enriched genes. (**b**–**g**) Number of up- and downregulated differentially expressed genes (DEGs) with the most special KEGG terms under different temperature treatments, and the specific KEGG terms include: “Lysosome” (**b**), “Amino sugar and nucleotide sugar metabolism” (**c**), “Fructose and mannose metabolism” (**d**), “Glycine, serine, and threonine metabolism” (**e**), “Neuroactive ligand–receptor interaction” (**f**), and “Glycolysis/gluconeogenesis” (**g**) pathways.

Notably, four metabolic pathways and one Environmental Information Processing pathway were significant changed in the T28 and T32 treatment groups (Figure 3a). In the metabolic pathways, DEGs belonging to “Amino sugar and nucleotide sugar metabolism”, “Fructose and mannose metabolism”, and “Glycine, serine and threonine metabolism” were significantly downregulated after T28 and T32 treatments, while those in the “Glycolysis/Gluconeogenesis” pathway were significantly upregulated (Figure 3c–f). In the “Environmental information-processing” pathway, DEGs belonging to the “Neuroactive ligand–receptor interaction” term were significantly upregulated after the T28 and T32 treatments (Figure 3g). Most DEGs implicated in these pathways were downregulated by temperature treatment (Figure 3b–e). However, only DEGs belonging to “Neuroactive ligand–receptor interaction” and “Glycolysis/gluconeogenesis” were induced by the T28 and T32 treatments, and most DEGs were upregulated at T32 (Figure 3f,g).

### 3.4. Overview of A. leii miRNA Sequencing Data

**Figure 4:**
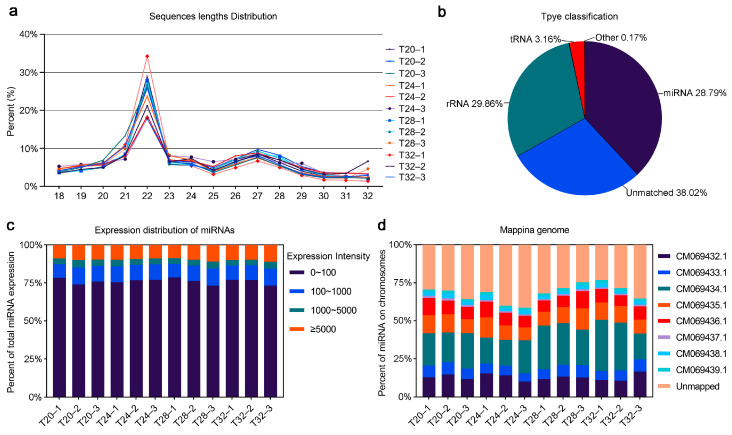
MicroRNAome of *A. leii* following various temperature treatments: (**a**) length distribution of all unique reads of all cleaned reads from 12 samples; (**b**) type classification for all sRNAs. The percentages of different RNA classes, including miRNA, tRNA, rRNA, unmatched, and other, are shown in a pie chart; (**c**) expression distribution of miRNAs in each sample examined in the array analysis; (**d**) distribution of miRNAs across different chromosomes in *A. leii*.

A MicroRNAome analysis was also conducted in *A. leii* under different temperatures (20 °C, 24 °C, 28 °C, and 32 °C). A total of 289,284,142 clean reads were obtained. The overall mapping rate for these reads was 60.19%, and quality scores at the levels of the mean Q20 and Q30 values of the clean data reached 99.19% and 97.30%, respectively (Table S8), indicating that the sequencing quality in this study was good. The sequence length distribution map shows that most of the clean reads were 18–24 nt in length, with 22 nt being the most common length (Figure 4a). A total of 61.98% of the clean reads were matched, annotated, and classified into different categories, including rRNA, tRNA, sRNA, snRNA, miRNA, and other Rfam RNA (Figure 4b). The rRNA and miRNA groups constituted the largest proportion of the samples, collectively representing 58.65% (Figure 4b). Only a quarter of the 1774 miRNAs examined were expressed at a significant level (with an intensity above 100). Out of these significantly expressed groups, about 13.83% of the miRNAs were expressed at levels between 1000 and 5000, and about 9.73% of the miRNAs were above 5000 (Figure 4c). The distribution of these miRNAs among the *A. leii* chromosomes is depicted in Figure 4d. Chromosome CM069432.1, CM069434.1, and CM069435.1 have high numbers of miRNA genes, almost up to 47%.

### 3.5. Analysis of DEMs of A. leii Under Different Temperature Treatments

**Figure 5:**
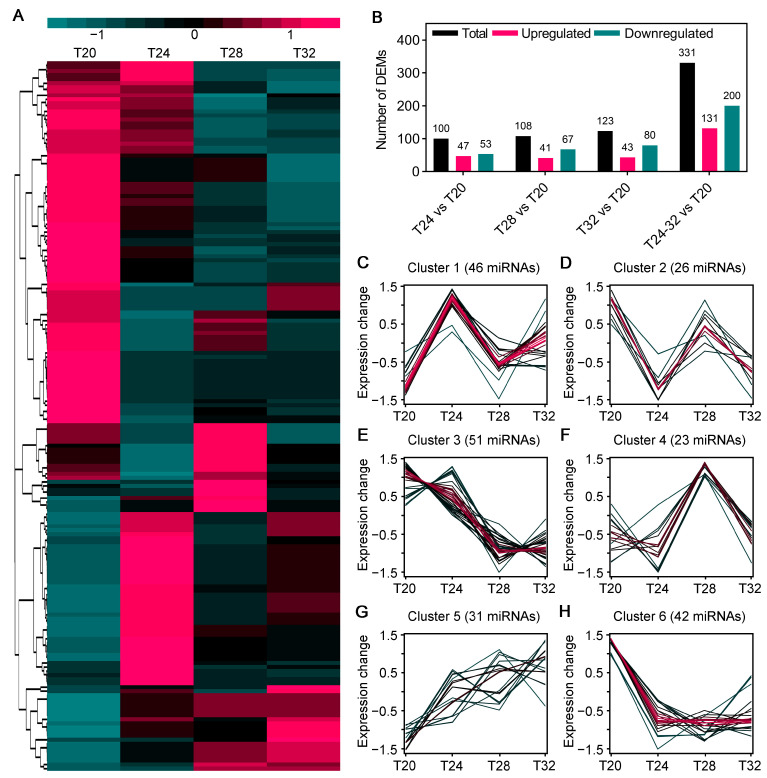
Analysis of DEMs under various temperature treatments in *A. leii*: (**A**) expression profile heatmap of DEMs identified in *A. leii* following temperature treatments at 20 °C, 24 °C, 28 °C, and 32 °C. The heatmap scale ranges represent normalized expression levels, from low to high, on a log2 scale from −1 to 1; (**B**) numbers of total, upregulated, and downregulated DEMs in *A. leii* after various temperature (20 °C, 24 °C, 28 °C, and 32 °C) treatments; (**C**–**H**) Mfuzz cluster analysis of differentially expressed DEMs by the C-means method. All identified DEMs are grouped into six clusters.

As the treatment temperature increased, the number of DEMs gradually rose, and the clustering heatmap of all DEMs showed that the miRNA levels were significantly different after various treatment temperatures (Figure 5A). A total of 100 DEMs were identified in the T24 vs. T20 comparison, including 47 upregulated and 53 downregulated DEMs; 108 DEMs in the T28 vs. T20 comparison, including 41 upregulated and 67 downregulated; and 123 DEMs were identified in the T32 vs. T20 comparison, with up- and downregulated DEMs of 43 and 80, respectively (Figure 5A). Temperature-responsive miRNAs were grouped into six clusters (Clusters 1–6) based on the similarity in their expression patterns (Figure 5C–H). Clusters 1 and 5 contained the most temperature-induced upregulated miRNAs, while Clusters 3 and 6 contained most temperature downregulated miRNAs (Figure 5C,G,E,H). Clusters 2 and 4 contained genes downregulated in T24, upregulated in T28 and downregulated in T32 (Figure 5D,F). Interestingly, temperature rapid responsive miRNAs belonged to Cluster 1, while temperature later responsive miRNAs belonged to Cluster 5 (Figure 5C,G).

### 3.6. Functional Annotation of DEM Targets of A. leii

As regulatory molecules, miRNAs execute diverse biological roles through post-transcriptional control of target genes. Computational prediction identified 2956 putative target genes collectively regulated by 220 miRNAs, consisting of 176 known miRNAs and 44 novel miRNAs. To identify the enriched GO and KEGG pathways, the *p*-values of the pathways enriched by their target genes were calculated using Fisher’s exact test. The GO classification revealed three dominant functional clusters. Within biological process, predominant annotations included “multicellular organism development” and “nervous system development” (*p* < 0.001) (Figure S4). The molecular function analysis highlighted “Ras guanyl-nucleotide exchange factor activity” and “Guanyl-nucleotide exchange factor activity” as key enzymatic activities (*p* < 0.001) (Figure S5). The cellular component showed predominant localization in “Neuron part” and “Plasma membrane part” (*p* < 0.001) (Figure S6). The KEGG enrichment analysis demonstrated maximal enrichment in “ECM–receptor interaction” and “Human papillomavirus infection” (*p* < 0.05) (Figure S7).

### 3.7. Analysis of Key Temperature-Responsive DEMs in A. leii

**Figure 6:**
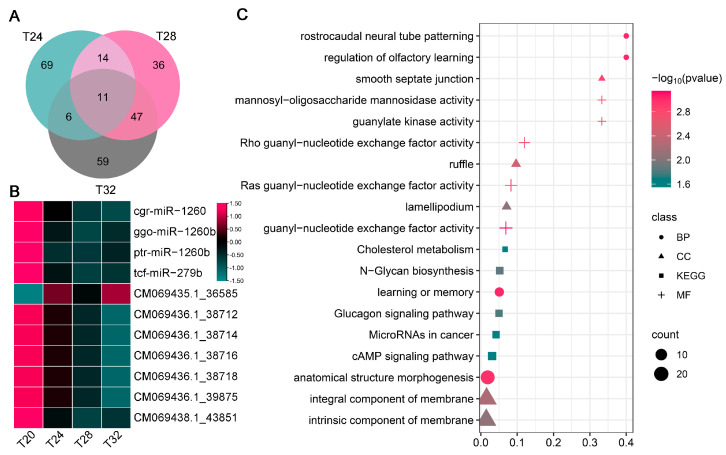
Analysis of key temperature-responsive DEMs under various temperature treatments in *A. leii*: (**A**) Venn diagram of the obtained DEMs; (**B**) heat maps of common DEMs between T24, T28, and T32; (**C**) GO and KEGG pathway enrichment analyses results of key temperature-responsive DEMs’ mRNA target genes. Biological process (BP), cellular component (CC), molecular function (MF), and KEGG represented, respectively, by dots, triangles, plus signs, and squares. The dot colors show the (−Log10(*p* value)) enrichment values, while the dot sizes reflect the number of genes in each enriched pathway.

We next grouped the common and conserved DEMs between the T24, T28 and T32 groups using a Venn diagram. A total of 11 common key DEMs were identified across the treatment groups (Figure 6A). Figure 6B presents a heat map of miRNA expression for the aforementioned DEMs. There were 585 interactions between 11 common key DEMs and 104 target genes for miRNAs (Table S9). In addition, we performed GO and KEGG enrichment analyses on the 104 target genes. The GO analysis shows that these target genes are primarily involved in memory, rostrocaudal neural tube patterning, ruffle, integral component of membrane, guanyl-nucleotide exchange factor activity, and Ras guanyl-nucleotide exchange factor activities (Figure 6C). Meanwhile, the KEGG pathway analysis revealed that these genes are primarily involved in N-glycan biosynthesis, glucagon signaling pathway, and microRNAs in cancer (Figure 6C). The analysis reveals that these GO terms and KEGG metabolic pathways may play roles in *A. leii*’s response to temperature.

### 3.8. Negatively Correlated of Key DEMs-DEGs Modules

**Figure 7:**
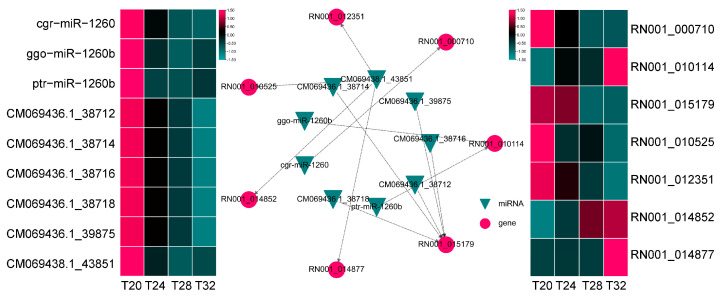
Expression profiles and interactions of important DEMs–DEGs under different temperature treatments. The middle section displays the negatively correlated modules of DEMs and DEGs. The heatmap on the left shows the expression of DEMs, while the heatmap on the right illustrates the expression of DEGs.

We constructed miRNA-mRNA interaction networks using key temperature-responsive DEMs and DEGs to identify the potential miRNAs and target genes correlated with high-temperature responsiveness. In total, 12 DEM-DEG pairs were identified, including *RN001_000710*, *RN001_010114*, *RN001_015179*, *RN001_010525*, *RN001_012351*, *RN001_014852*, and *RN001_014877*, and miRNAs negatively regulate the expression of their target genes (Figure 7). Among these pairs, two mRNA genes (*RN001_014852* and *RN001_014877*) were found to be a target of CM069438.1_43851 and upregulated in *A. leii* with the increase in temperature. In addition, *RN001_010114*, the target gene of ggo-miR-1260b and ptr-miR-1260b, showed significantly increased expression levels with rising temperature. These identified DEMs-DEGs pairs were considered as candidate miRNA-mRNA pairs for high-temperature-responsive genes in *A. leii*.

### 3.9. Validation of the 70-Kilodalton Heat Shock Protein (HSP70) Gene by qPCR

**Figure 8:**
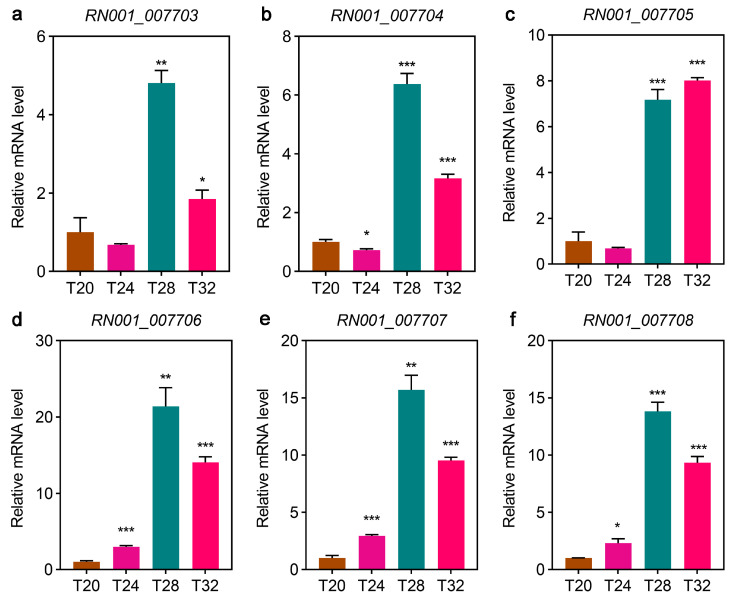
Quantitative PCR (qPCR) data for the mRNA expression profiles of the 70-kilodalton heat shock protein (*HSP70*) gene of *A. leii* under different temperature treatments. The T20 group’s mRNA levels were set at 1.0 units, and other treatment group values are expressed relative to them. The data are presented as the mean ± standard deviation (SD) of three biological replicates. Asterisks indicate significant differences (* *p* < 0.05, ** *p* < 0.01, and *** *p* < 0.001).

The expression levels of six temperature-responsive genes of *HSP70* were analyzed using qPCR. The expression levels of *RN001_007703*, *RN001_007704*, *RN001_007706*, *RN001_007707*, and *RN001_007708* significantly increased in the T24 and T28 groups but decreased in the T32 group in *A. leii* (*t*-test, *p* < 0.05) (Figure 8). Notably, the expression levels of RN001_007705 significantly increased with an increase in temperature (*p* < 0.05) (Figure 8c). The mRNA expression levels of these six *HSP70* genes were consistent with the RNA sequencing results (Figure 8), thus validating the reliability of the RNA sequencing data and the computational methods we used.

## 4. Discussion

The gradual increase in global temperatures, along with the increased frequency, intensity, and duration of extreme high-temperature events, is leading to serious impacts on production performance and significant threats to insect populations [3,6,59,60]. Nevertheless, the capacity of insects to mitigate high-temperature stress has been subject to ongoing evolution in different aspects, including physiological, biochemical, cellular, and behavioral mechanisms to counteract high temperatures [13,14]. With the development of high-throughput sequencing technology, the integrated analysis of multi-omics has been widely used in studies of insect growth, development, immunity, biological and abiotic stresses, etc. [61,62]. In this study, we employed RNA-seq and miRNA-seq methods to conduct a combined analysis of mRNA and miRNA transcriptomes, aiming to better elucidate the molecular mechanisms underlying the temperature-dependent responses of *A. leii* larvae.

As anticipated, the number of DEGs in *A. leii* larvae increased in tandem with the temperature increase, as revealed by the mRNA dynamic expression analysis under different temperature treatments. Specifically, we identified 2841 DEGs at T24, T28, and T32, including 1369 upregulated genes and 1472 downregulated genes (Figure 2a). Herein, several *HSPs* were brought to our attention. Heat stress proteins are central response factors in insect heat tolerance studies, and upregulation of their expression stabilizes denatured proteins and repairs cellular damage [16,17]. In *A. leii*, there are 14 temperature-responsive *HSPs*, mainly from the *HSP70* and *sHSP* families. Seven of the *HSPs* reached their highest expression at T28 and the other seven at T32, which may be a way for *A. leii* to adapt to extreme environments by retaining the ability to consistently expressing specific *HSPs*. The same trend was also found in the yellow fever mosquito [63]. Furthermore, KEGG analysis showed that DEGs of T24, T28, and T32 were commonly enriched in some pathways, including “Metabolic pathways”, “Biosynthesis of amino acids”, “Retinol metabolism”, and “Lysosome” (Figure 3a). This outcome aligns with our initial hypothesis, as insects have developed a range of adaptative metabolic strategies to manage high temperatures [13,16]. Our results indicate that the metabolism of *A. leii* larvae at high temperatures was markedly weaker, and this suppression of metabolic progress may be a conservative mechanism reflecting cellular homeostasis or a means of conserving energy to cope with high temperatures, as observed in *Glyphodes pyloalis* [64]. It is worth noting that some amino acid metabolism and carbohydrate metabolism pathways belonged to the T28 and T32 groups only, including “Amino sugar and nucleotide sugar metabolism”, “Fructose and mannose metabolism”, “Glycine, serine, and threonine metabolism”, and “Glycolysis/gluconeogenesis” (Figure 3a). It is possible that this is due to the elevated temperatures in the T28 and T32 groups, which requires *A. leii* larvae to utilize a large numbers of genes involved in carbohydrate metabolism to maintain normal life activities. The mean temperature has been shown to strongly influence amino acid and carbohydrate metabolism in insects, with metabolism increasing at higher temperatures [65]. This response likely serves as a compensatory mechanism to maintain proper energy regulation and reflects the direct impact of temperature on metabolic activity in *A. leii* at 28  °C [65]. Moreover, we discovered that Neuroactive ligand–receptor interaction of Environmental Information Processing was significantly enriched in T24 and T28 group (Figure 3a). The Neuroactive ligand–receptor interaction signaling pathway is directly associated with neurological function. Neuroactive ligands modulate neuronal activity by binding to intracellular receptors, which possess the ability to bind transcription factors and regulate gene expression [66,67]. Research has shown that in *Apis cerana* and *Apis mellifera* infested by *Varroa destructor*, exposed to the neonicotinoids in *Chironomus dilutus*, and exposed to the volatile pesticide dichlorvos in *Spodoptera litura*, the neuroactive ligand–receptor interaction pathway is significantly enriched [66,68,69]. The above evidence suggests when insects are stimulated by their environment, the relevant signaling pathways in their cells are activated. Therefore, we speculate that *A. leii* larvae begin to respond when the temperature exceeds 28 °C, involving multiple aspects such as neural signaling, physiological responses, and adaptive regulation.

miRNA are important regulatory molecules that participate in growth, development, environmental adaptability, and stress resistance in animals and plants [34,70]. At present, many miRNAs have been reported to respond to environmental stress signals in insects, such as miR-31-5p, mmu-miR-3475-3p, and miR-277 [34,71,72]. These miRNAs play key roles in modulating cellular responses to stress. In *Monochamus alternatus*, miR-31-5p modulates cold acclimation through ascaroside (asc-C9) by inhibiting acyl-CoA oxidase, the rate-limiting enzyme in peroxisomal β-oxidation cycles [71]. mmu-miR-3475-3p were involved in the response of *Lymantria dispar* to cyantraniliprole stress by regulating five genes associated with detoxification [72]. In *A. leii*, miR-277 regulates several target genes that are typically related to reactive oxygen species accumulation and DNA damage, playing crucial roles in response to benzo[a]pyrene exposure [34]. In our study, 220 DEMs were identified in *A. leii* under different temperature treatments, including 176 known and 44 novel miRNAs, and 2956 target genes of the DEMs were predicted. Our analysis of miRNA omics revealed that miRNA expression in *A. leii* under temperature treatments was significantly downregulated (Figure 5B). This reduction may have led to an increase in mRNA release, thereby increasing the production of environment-specific proteins, preventing the cellular structures damage of protect against or repair [73]. Additionally, it may activate DEGs that assist *A. leii* in adapting to high temperature. Figure 7 demonstrated that miRNAs target multiple genes and genes were affected by the downregulation of multiple miRNAs. Among them, *RN001_010114* (SLC29A4) is an equilibrative nucleoside transporter that may catalyze the transport of adenosine, monoamine and xenobiotics. The upregulation of SLC29A4 may increase the provision of nucleosides, enhance metabolite flux, and alter metabolic exchange, as well as increase energy metabolism [74]. The increased expression of this gene may serve to counteract the reduction in the expression of other genes within the metabolic pathway, thereby maintaining metabolic balance, which was consistent with the findings from transcriptomic analysis. A novel miRNA of CM069438.1_43851, was identified as targeting five DEGs. Among these, upregulations of *RN001_014852* and *RN001_014877* were observed. In insects, *RN001_014852* (trypsin) is released by the midgut and plays key roles in digestion and activation of other zymogens [75]. In this study, the gene encoding *RN001_014852* (trypsin) levels were lower in the T20 and T24 groups and increased in the T28 and T32 groups, indicating that *RN001_014852* may be an important response gene of high-temperature (Figure 7). In addition, *RN001_014877* (the gene encoding AAA domain) levels were sharply induced by high temperatures of 32 °C (Figure 7). These research outputs suggest that CM069438.1_43851 may be a novel temperature response miRNA, and reveal that CM069438.1_43851 negatively regulates its target gene of the gene encoding trypsin and encoding the AAA domain.

In this study, all individual *A. leii* used were sixth instar larvae, and although there was some variation in their life cycles and feeding behavior, the developmental effects of individual differences were kept to a minimum. Overall, our results reveal the significant effects of temperature change on the metabolic activities of *A. leii*, especially in amino acid and carbohydrate metabolism. These findings are not only important for understanding physiological adaptation mechanisms and artificial feeding of this species, but also provide important references for the conservation of *A. leii* and other aquatic insects in the context of climate change. The results of this study are somewhat limited despite the strict temperature-controlled treatments. High temperatures can often bring about other stresses, such as the fact that warm waters are more likely to deoxygenate or that warm water can increase the respiration rate of aquatic insects, thus limiting their ability to cope with low levels of dissolved oxygen [19]. However, for some DEGs and DEMs, whether it is a temperature response or a hypoxia response cannot be directly determined.

## 5. Conclusions

In conclusion, we combined miRNA and mRNA datasets to uncover the regulatory network involved in response to high temperatures of *A. leii*, resulting in the identification of 1983 DEGs and 220 DEMs. In addition, some known and novel miRNAs with their target genes were predicted as ideal candidates for future manipulation to improve high-temperatures tolerance. In summary, the identified miRNA-mRNA pairs may enhance our understanding of transcriptional regulation related to tolerance traits in the aquatic firefly *A. leii* when exposed to high temperatures. This knowledge provides deeper insight into the molecular responses to high temperature in *A. leii* at the miRNA and mRNA levels. Despite providing initial mRNA and miRNA profiles for high-temperature responses in *A. leii*, these results are preliminary and need to be further verified experimentally. In addition, results from a small number of samples are limited and may reduce statistical power. Consequently, additional experiments are essential to fully explore the high-temperature resistance mechanism, and we recommend that more studies be conducted in this area.

## Data Availability

The data presented in this study are available upon request from the corresponding author.

## Supplementary Materials

The following supporting information can be downloaded at: https://www.mdpi.com/article/10.3390/insects16030316/s1, Figure S1: The top 15 enriched KEGG pathways of DEGs under the T24 treatment; Figure S2: The top 15 enriched KEGG pathways of DEGs under the T28 treatment; Figure S3: The top 15 enriched KEGG pathways of DEGs under the T32 treatment; Figure S4: The biological process of GO enrichment analysis of DEM target genes of *A. leii* following temperature treatments; Figure S5: The molecular function from the GO enrichment analysis of DEM target genes in *A. leii* following temperature treatments; Figure S6: The cellular components from the GO enrichment analysis of DEM target genes in *A. leii* following temperature treatments; Figure S7: KEGG pathway enrichment of target mRNAs; Table S1: Firefly habitat characteristics; Table S2: List of primers used in this study; Table S3: Summary of the RNA-sequencing of *Aquatica leii* exposed to different temperatures; Table S4: Classification information of the annotated GO terms from the RNA-sequencing of *Aquatica leii* exposed to different temperatures; Table S5: GO enrichment analysis of the DEGs of *Aquatica leii* exposed to a temperature of 24 °C; Table S6: GO enrichment analysis of the DEGs of *Aquatica leii* exposed to a temperature of 28 °C; Table S7: GO enrichment analysis of the DEGs of *Aquatica leii* exposed to a temperature of 32 °C; Table S8: Summary of microRNAome of *Aquatica leii* exposed to different temperatures; Table S9: List of miRNA–mRNA interactions in *Aquatica leii* exposed to different temperatures.
