# Supplementary material for: Integrated mRNA and miRNA Omics Analyses Reveal Transcriptional Regulation of the Tolerance Traits by Aquatica leii in Response to High Temperature
Source: Insects. 2025 Mar 18;16(3):316. doi: 10.3390/insects16030316 (PMC11943311; doi:10.3390/insects16030316)
Supplement: Supplementary file 1 [file insects-16-00316-s001.zip › Supplement figure.docx]

**Figure.S1**


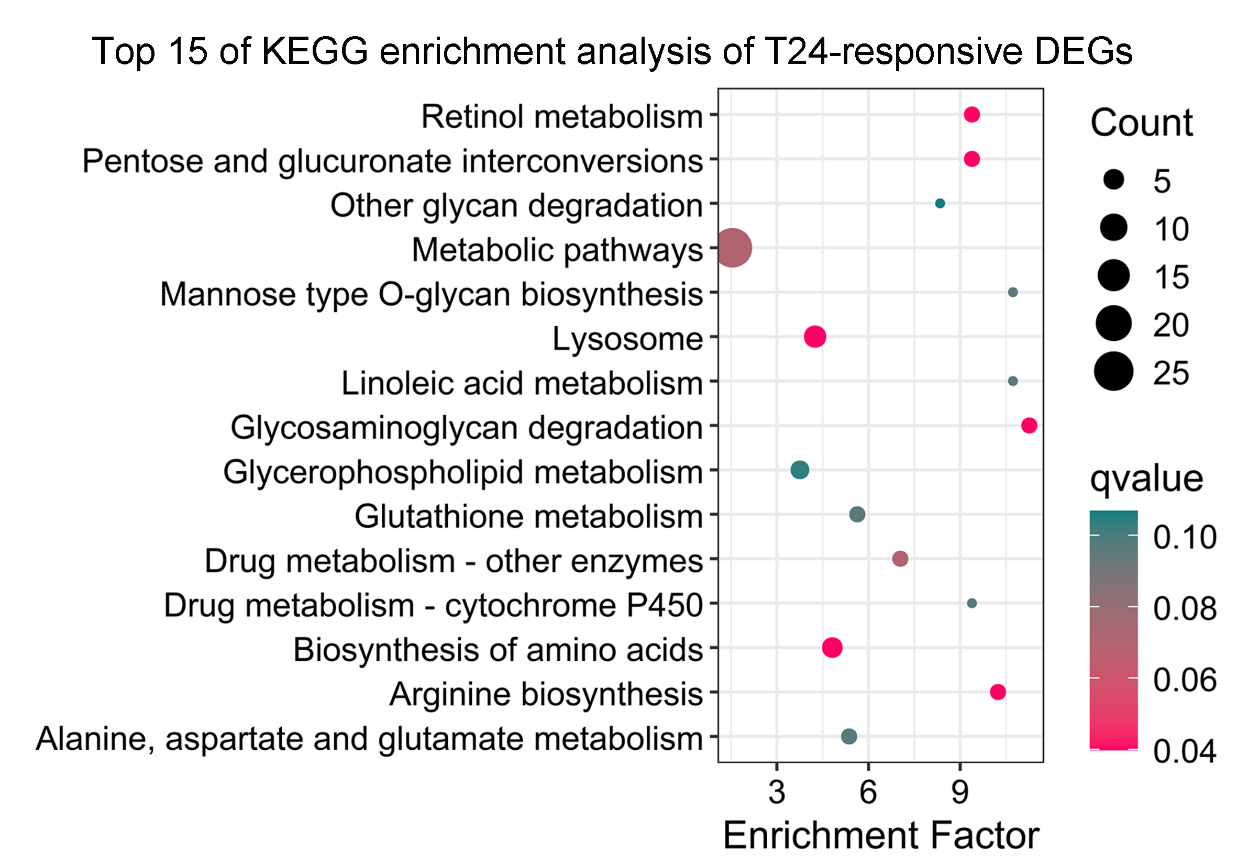


Figure.S1. The top 15 enriched KEGG pathways of DEGs under the T24 treatment. The dot colors indicate the qvalue enrichment values, and the dot sizes represent the number of genes within each enriched pathway.

**Figure.S2**


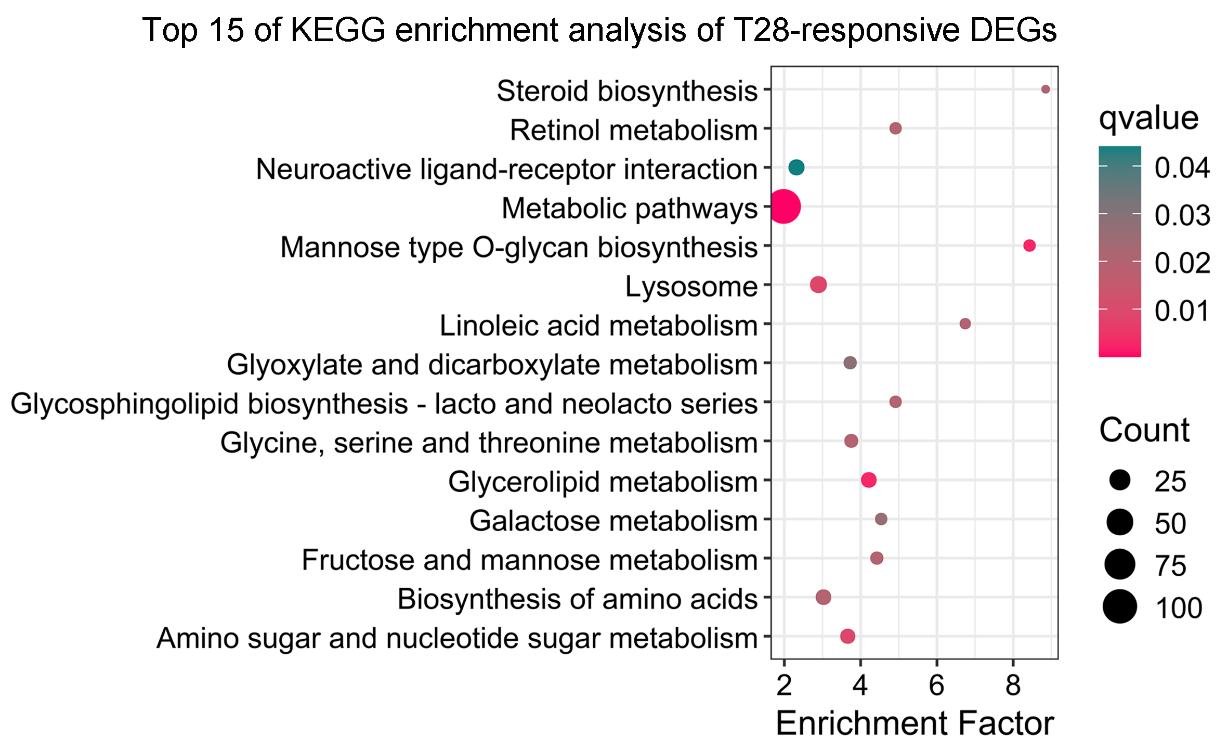


Figure.S2. The top 15 enriched KEGG pathways of DEGs under the T28 treatment. The dot colors indicate the qvalue enrichment values, and the dot sizes represent the number of genes within each enriched pathway.

**Figure.S3**


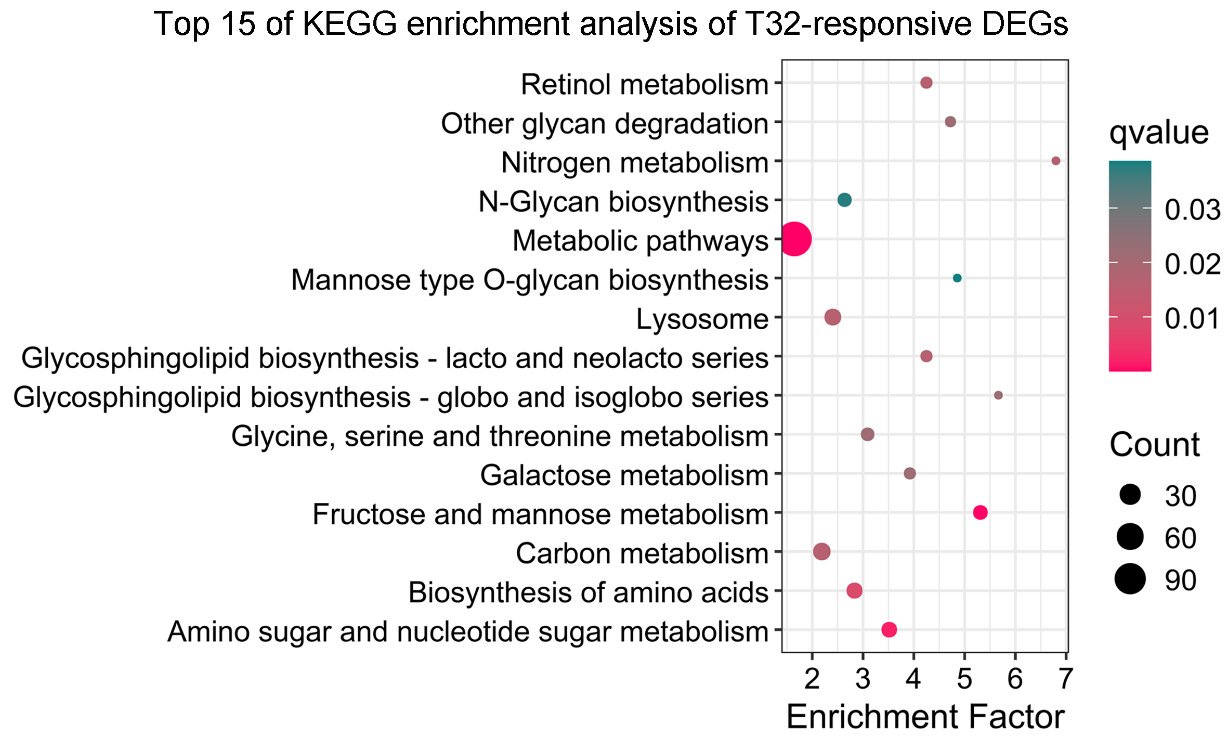


Figure.S3. The top 15 enriched KEGG pathways of DEGs under the T32 treatment. The dot colors indicate the qvalue enrichment values, and the dot sizes represent the number of genes within each enriched pathway.

**Figure.S4**


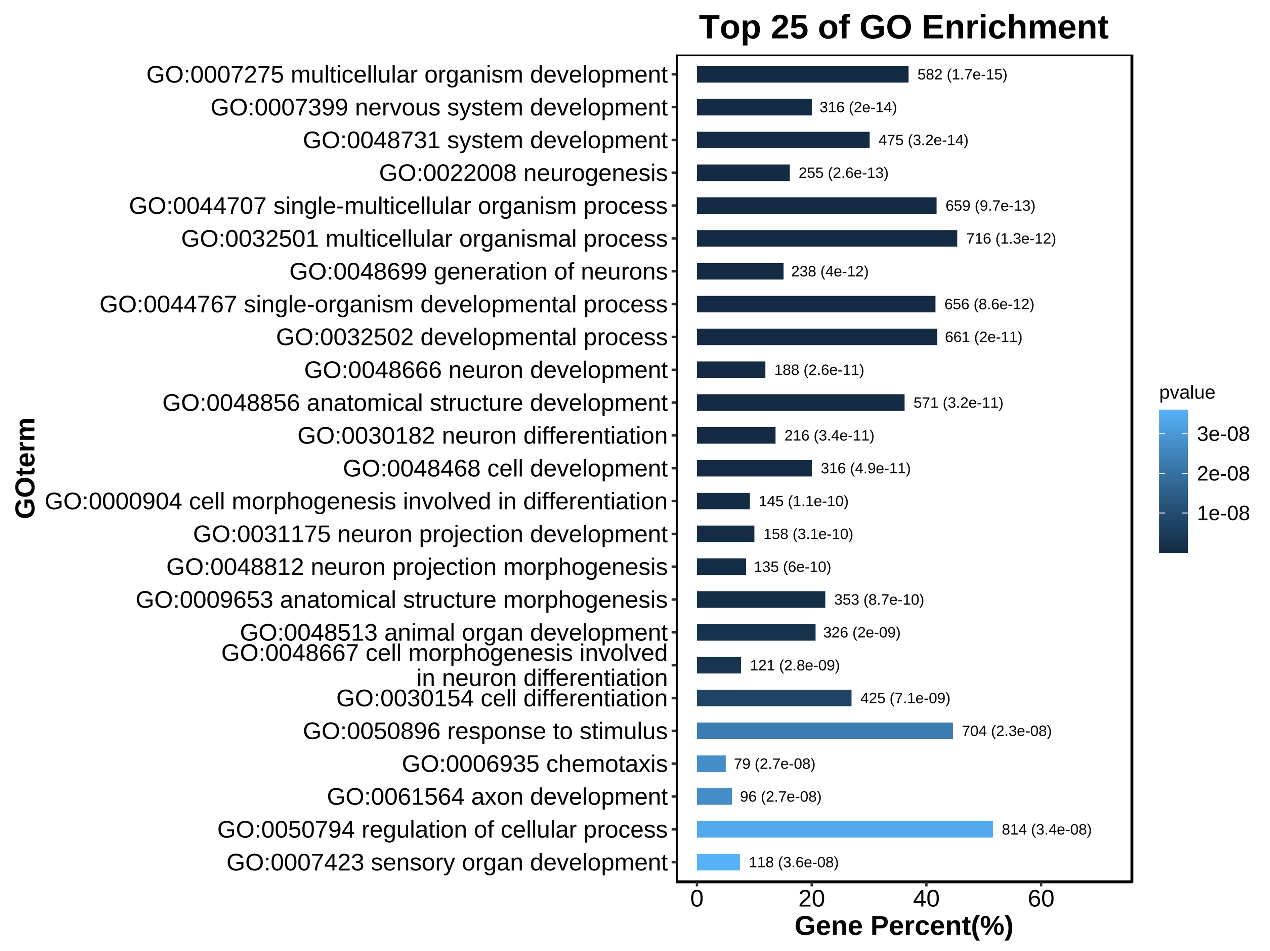


Figure.S4. The biological process of GO enrichment analysis of DEM target genes of *A. leii* following temperature treatments. The x-axis shows the Gene Percent (%), and the y-axis lists the GO terms. Each bar represents the gene percentage of a GO term, with color intensity reflecting the p-value—darker shades indicate higher significance. Specific p-values for each GO term are labeled in the figure.

**Figure.S5**


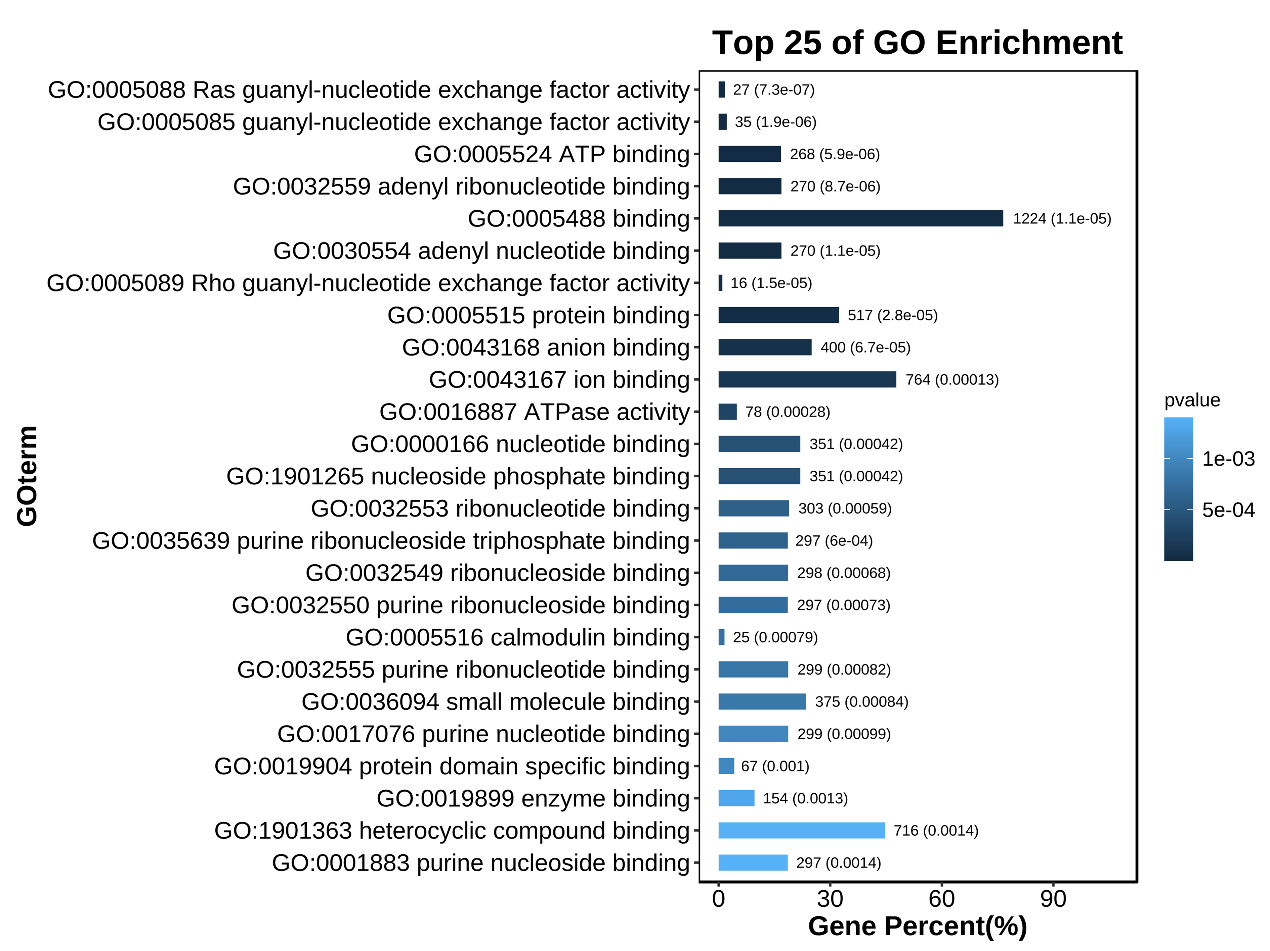


Figure.S5. The molecular function from the GO enrichment analysis of DEM target genes in *A. leii* following temperature treatments. The x-axis shows the Gene Percent (%), and the y-axis lists the GO terms. Each bar represents the gene percentage of a GO term, with color intensity reflecting the p-value—darker shades indicate higher significance. Specific p-values for each GO term are labeled in the figure.

**Figure.S6**


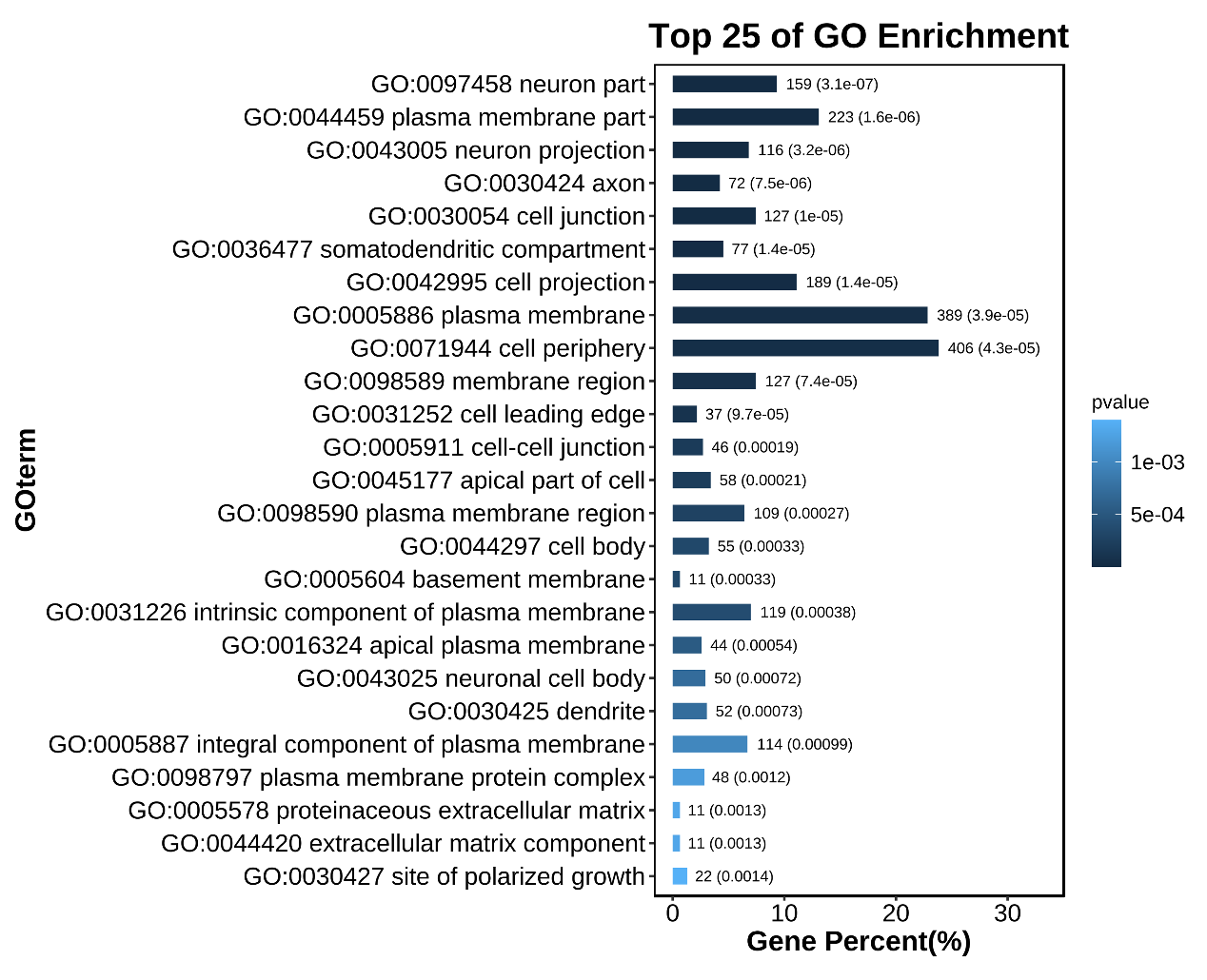


Figure.S6. The cellular components from the GO enrichment analysis of DEM target genes in *A. leii* following temperature treatments. The x-axis shows the Gene Percent (%), and the y-axis lists the GO terms. Each bar represents the gene percentage of a GO term, with color intensity reflecting the p-value—darker shades indicate higher significance. Specific p-values for each GO term are labeled in the figure.

**Figure.S7**


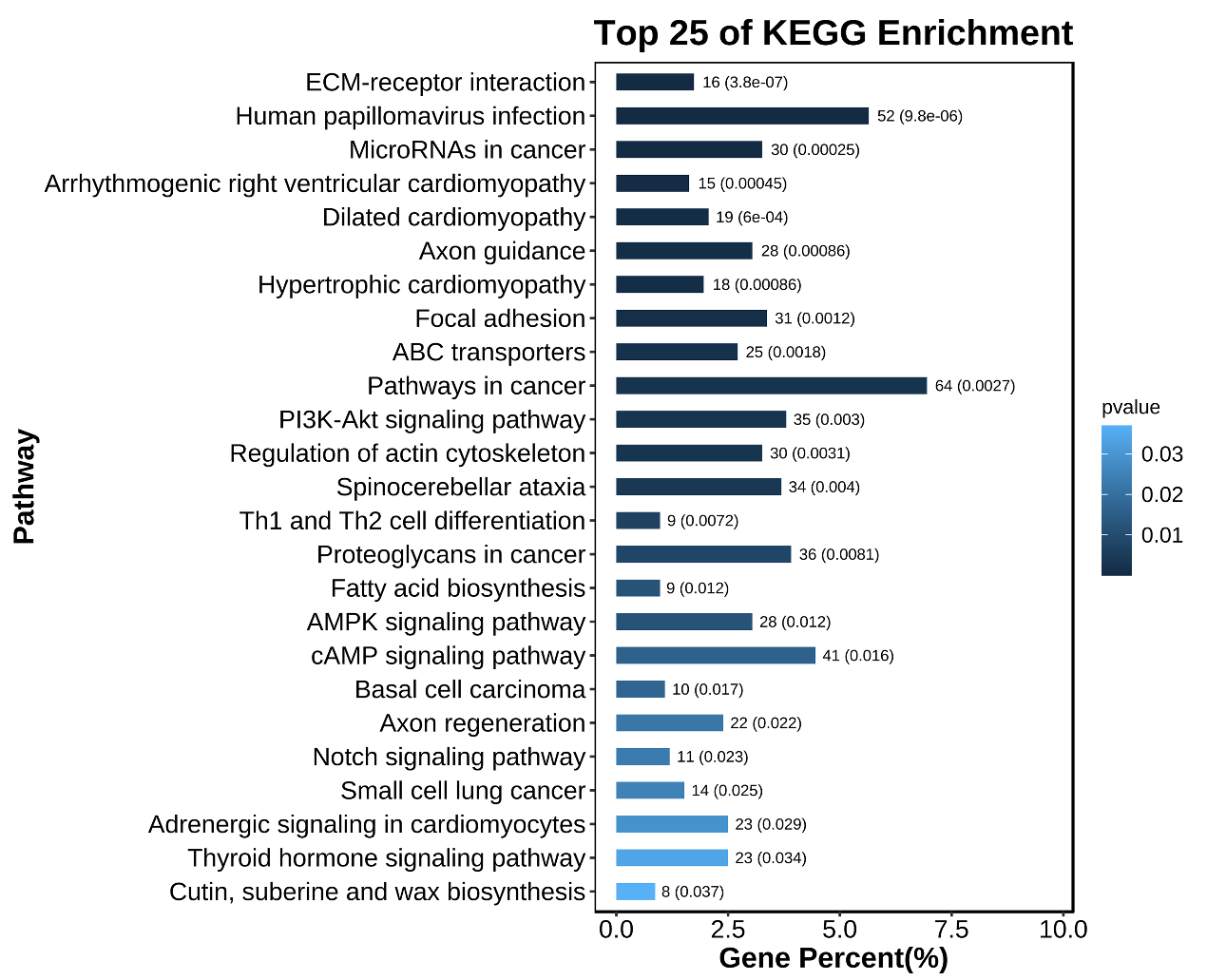


Figure.S7. KEGG pathway enrichment of target mRNAs. This figure presents the top 25 KEGG pathway enrichment results. The x-axis shows the Gene Percent (%), and the y-axis lists the GO terms. Each bar represents the gene percentage of a GO term, with color intensity reflecting the p-value—darker shades indicate higher significance. Specific p-values for each GO term are labeled in the figure.
